# Influence of non-uniform magnetic field on the thermal efficiency hydrodynamic characteristics of nanofluid in double pipe heat exchanger

**DOI:** 10.1038/s41598-022-26285-w

**Published:** 2023-01-09

**Authors:** Y. Azizi, M. Bahramkhoo, A. Kazemi

**Affiliations:** Department of Mechanical Engineering, Bandar Anzali Branch, Islamic Azad University, Bandar Anzali, Iran

**Keywords:** Mechanical engineering, Nanoscale materials

## Abstract

Enhancement of the heat transfer rate inside the double pipe heat exchangers is significant for industrial applications. In present work, the usage of non-uniform magnetic field on the heat transfer rate of the nanofluid flow streamed inside double pipe heat exchangers are comprehensively studied. Computational technique of CFD is used for the visualization of the nanofluid hydrodynamic in existence of the magnetic source. Influences of the magnetic intensity and nanofluid velocity on the heat transfer are also presented. Simple algorithm is used for the modeling of the incompressible nanofluid flow with addition of magnetic source. Presented results show that magnetic source intensifies the formation of the circulation in the gap of the inner tube and consequently, heat transfer is enhanced in our domain. Comparison of different geometries of tube reveals that the triangle tube is more efficient for improvement of the heat transfer of nanofluid flow. Our results indicate that heat transfer in the tube with triangular shape is more than other configurations and its performance is 15% more than smooth tube.

## Introduction

The management of the heat transfer process is significant for the development of recent engineering and industrial systems and devices^1,2^. There are several techniques and materials for isolation have been used and presented in recent years. Although heat transfer reduction is easily accessible by using isolators, heat transfer improvement is not easily achievable due to limitations in materials. Meanwhile, heat transfer improvement is more required in industrial and engineering instruments and devices i.e. heat exchangers, and condensers^3,4^. The importance of efficient heat transfer has motivated mechanical engineers and researchers to find new solutions and materials which increase thermal transfer in industrial applications^5^.

The application of fin is the most conventional approach which is widely used due to its simplicity and low cost. In this methodology, the contact surface area of the heat source with the outside is increased by adding a fin adjacent to a heat source^6,7^. Although several papers investigated this technique for the heat transfer rate, the efficiency of the heat transfer via fin is limited. The shape effects are also considered an old-fashion method for heat transfer enhancement^8–10^.

The main revolutionary in heat transfer is achieved by the addition of nanoparticles to the base fluid. In fact, the existence of the Ferro particles inside the main fluid extensively augments due to Ferro characteristics of the fluid mixture^11^. The addition of Ferro Nano-particles improves the heat capacity and thermal conductivity of the fluid mixture and this increases the heat transfer efficiency in heat exchangers in real applications^12,13^. Theoretical investigations of the nanofluid heat transfer have been widely done to obtain efficient condition. In the last decades, the advance in computational fluid dynamics enables scholars to model and simulate nano heat transfer modeling in complex and real industrial devices^14,15^. These researches have presented significant results about the mechanism of heat transfer of base fluid with nano Ferro particles in different processes in melting and boiling phenomena. They also investigated phase change materials PCM via CFD methods with/without nanoparticles^16,17^. These investigations have revealed various aspects of the nanofluid in industrial usage^18^.

**Figure 1:**
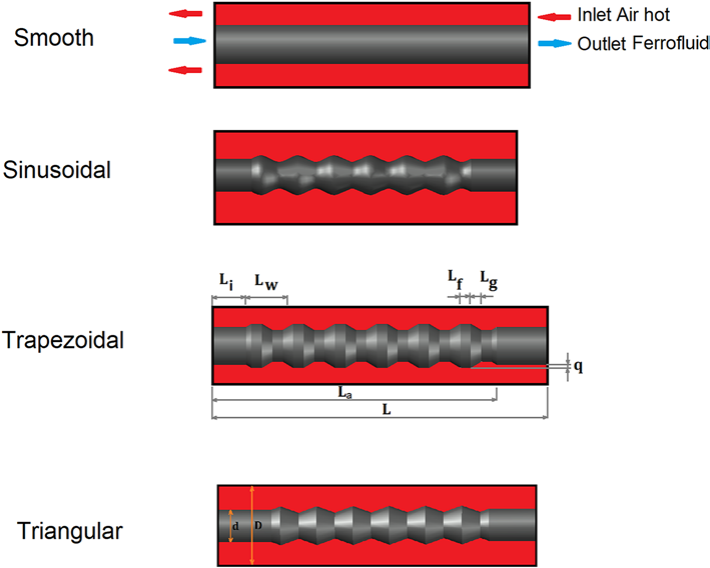
Investigated models.

The application of the magnetic field also considerably boosts the heat transfer of the ferrofluid due to exerted force on Ferro particles of the nanofluid stream^19,20^. This type of problem is mainly divided into two main parts: uniform and non-uniform magnetic fields. Although the efficiency of the uniform magnetic field is more non-uniform, the production of the uniform magnetic field is an almost challenging task and requires enough space. Besides, its cost is more than the non-uniform magnetic field which is obtained via the existence of the wire with AC/DC current. With the simplicity and lower cost of the non-uniform magnetic fields in industrial applications, this topic is attractive in thermal engineering science^21,22^. The experimental investigations of the non-uniform magnetic fields have been presented in limited research since the measurement technique of heat transfer in this specific condition is a tough task^23–26^. Unlike a uniform magnetic fields, the simulation of the non-uniform magnetic source requires high skill for the implementation of the source term in the main governing equations in the modeling process^27–30^. There are limited investigations that reported the ferrofluid stream in the existence of the non-uniform magnetic field. In this study, the simulation of the water stream with nanoparticles is investigated in the existence of the non-homogeny magnetic field as displayed in Fig. 1.

As displayed in Fig. 1, the Ferrofluid is used as a coolant for heat transfer from the hot air stream moving in outside tube. In the hot stream, there is a wire which is the source of the magnetic field for heat transfer improvement inside the ferrofluid. This study tries to visualize the impacts of the non-uniform source of magnetic field on the heat transfer performance of shell and tube heat exchangers. Although this type of heat exchanger is the most convenient one and has been widely investigated^31–35^, the performance of this type under the impact of the non-homogenous magnetic field was not fully discussed.

In present work, comprehensive researches are done to disclose heat transfer efficiency of ferrofluid in the presence of the magnetic source near the shell and tube heat exchangers. Computational approach of CFD is used for the simulation of the hydrodynamic and thermal characteristics of ferrofluid in different operational condition. The influences of magnetic source and ferrofluid velocity on the heat transfer efficiency are presented. The different geometries of the inner tube are also investigated in this article. Temperature variation of ferrofluid along the tube is demonstrated and compared in various conditions.

## Governing equations and numerical procedures

**Figure 2:**
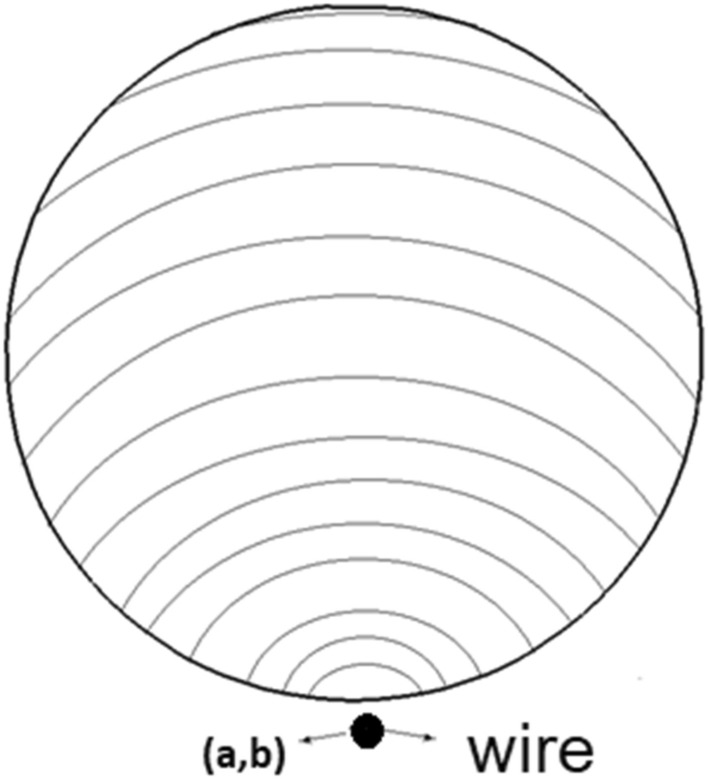
Distribution of non-uniform magnetic field by wire.

Figure 1 illustrates the schematic view of selected model for our investigation. As presented in this figure, the hot air transfers from the outer domain (shell) while ferrofluid is moved counter currently from inner tube (d). There is a wire inside the shell to produce magnetic field for heat transfer improvements. Different shapes of tube are investigated in the present work. To achieve reliable results, 3-D models of selected geometries are chosen. The existence of wire generates the non-uniform magnetic field as displayed in Fig. 2. In the subsequent section, the influence of the magnetic fields on the flow structure would be explained in details.

**Figure 3:**
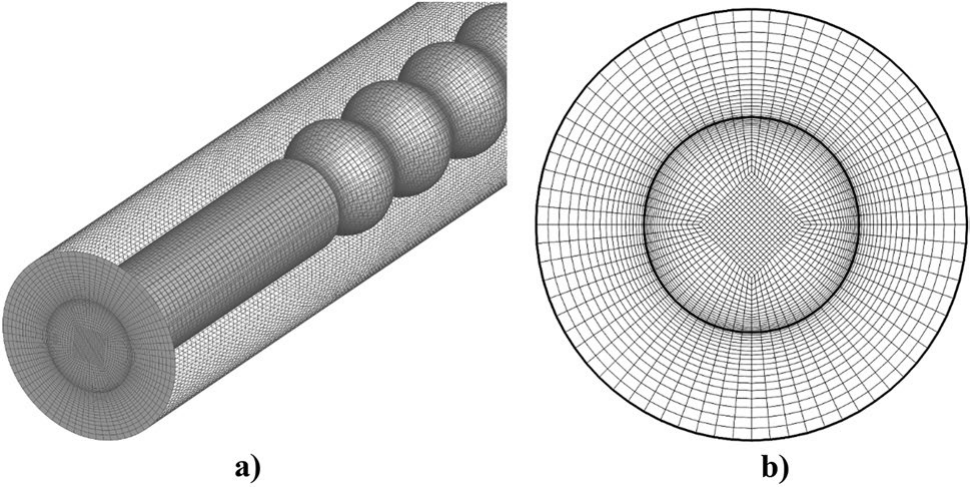
Applied grids (**a**) 3-D view, (**b**) cross section.

**Figure 4:**
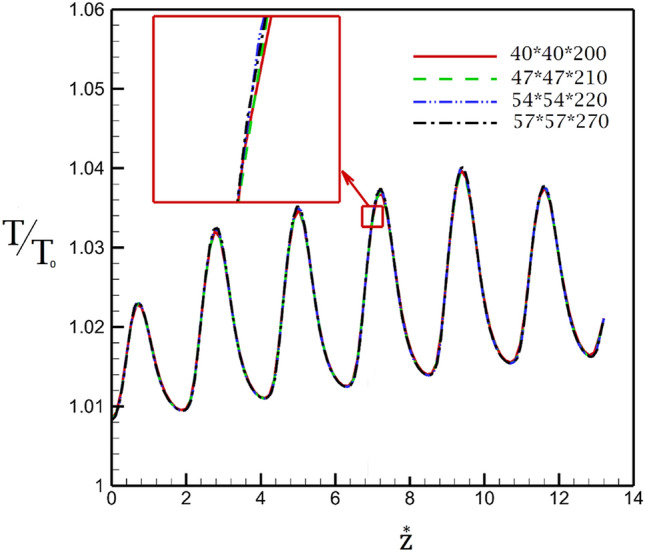
Grid study.

Figure 3 demonstrated the applied grids for the selected models. The structured grid is produced in the chosen 3-D models as displayed in this figure^22,23,36^. Grid studies are also done by examining various grid size and resolutions and results of non-dimensional temperature are compared in Fig. 4. Presented results indicate that the fine grid (54 × 54 × 220) is acceptable for the future investigations.

Since the base fluid for the nanofluid is water, solving RANS equations with energy equations would results in reasonable results^34–37^. For the simulation of the magnetic field, the components of the magnetic field must be added in the source term in the momentum equations. It is supposed that the impacts of magnetic fields on the nanofluid properties are minor, and the Lorentz force is not substantial in the momentum equations in comparison with the magnetic force by reason of the electrical conductivity. Hence, the ultimate equations for our models are as follows:

1 $$\frac{\partial u}{\partial x}+\frac{\partial v}{\partial y}+\frac{\partial w}{\partial z}=0$$

2 $${\rho }_{m}\left(u\frac{\partial u}{\partial x}+v\frac{\partial u}{\partial y}+w\frac{\partial u}{\partial z}\right)=- \frac{\partial p}{\partial x}+{\mu }_{m} \left(\frac{{\partial }^{2}u}{{\partial x}^{2}}+\frac{{\partial }^{2}u}{{\partial y}^{2}}+\frac{{\partial }^{2}u}{{\partial z}^{2}} \right)+{F}_{K}(x)$$

3 $${\rho }_{m}\left(u\frac{\partial v}{\partial x}+v\frac{\partial v}{\partial y}+w\frac{\partial v}{\partial z}\right)= - \frac{\partial p}{\partial y}+{\mu }_{m} \left(\frac{{\partial }^{2}v}{{\partial x}^{2}}+\frac{{\partial }^{2}v}{{\partial y}^{2}}+\frac{{\partial }^{2}v}{{\partial z}^{2}} \right)+{F}_{K}(y)$$

4 $${\rho }_{m}\left(u\frac{\partial w}{\partial x}+v\frac{\partial w}{\partial y}+w\frac{\partial w}{\partial z}\right)= - \frac{\partial p}{\partial z}+{\mu }_{m} \left(\frac{{\partial }^{2}w}{{\partial x}^{2}}+\frac{{\partial }^{2}w}{{\partial y}^{2}}+\frac{{\partial }^{2}w}{{\partial z}^{2}}\right)$$

5 $${\left({\rho }_{m}{C}_{p}\right)}_{m}\left(u\frac{\partial T}{\partial x}+v\frac{\partial T}{\partial y}+w\frac{\partial T}{\partial z}\right)={ k}_{m} \left(\frac{{\partial }^{2}T}{{\partial x}^{2}}+\frac{{\partial }^{2}T}{{\partial y}^{2}}+\frac{{\partial }^{2}T}{{\partial z}^{2}} \right)$$

The term ${F}_{K}\left(x\right)={\mu }_{0}M\frac{\partial H}{\partial x}$ and ${F}_{K}\left(y\right)={\mu }_{0}M\frac{\partial H}{\partial y}$ are component of Kelvin force signify the incidence of the magnetic gradient in chosen domain. are the components of Kelvin body force in the x and y directions, respectively. H_x_, H_y_ are the components of the magnetic field in the x and y directions are determined as follows:

In present, the SIMPLEC algorithm is used with the second order upwind numerical scheme^38–40^. This algorithm is a normally used in Computational Fluid Dynamics for solving the very Navier–Stokes equation’s. The algorithm follows the same steps like the SIMPLE algorithm with a little variation that the momentum equations are manipulated which allows SIMPLEC velocity correction equations to omit terms that are less significant than those omitted in SIMPLE. Moreover, finite volume computational fluid dynamic code is used to resolve governing equations and the facts of the code are completely clarified in preceding articles.

Applied boundary condition for the chosen model is also displayed in Fig. 1. The inlet velocity of ferrofluid is equivalent to the Reynolds number = 80, 100 and 120. The air stream velocity is equivalent to the Re = 1500. The properties of the ferrofluid, air and gas are presented in Table 1. Fe304 nanoparticles with 4% concentration is mixed with base fluid for the production of the ferrofluid.

**Table 1:** Thermo properties.

	$\mu (\mathrm{kg}/\mathrm{m s})$	$\mathrm{k }(\mathrm{w}/\mathrm{mk})$	$cp (\mathrm{J}/\mathrm{kg k})$	$\rho (\mathrm{kg}/{\mathrm{m}}^{3})$
$\left(\mathrm{p}\right) {\mathrm{Fe}}_{3}{\mathrm{O}}_{4}$	–	6	670	5200
Water $\left(f\right)$	0.001003	0.6	4182	998.2
Gas	0.00001087	0.033	–	0.6679

## Results and discussion

**Figure 5:**
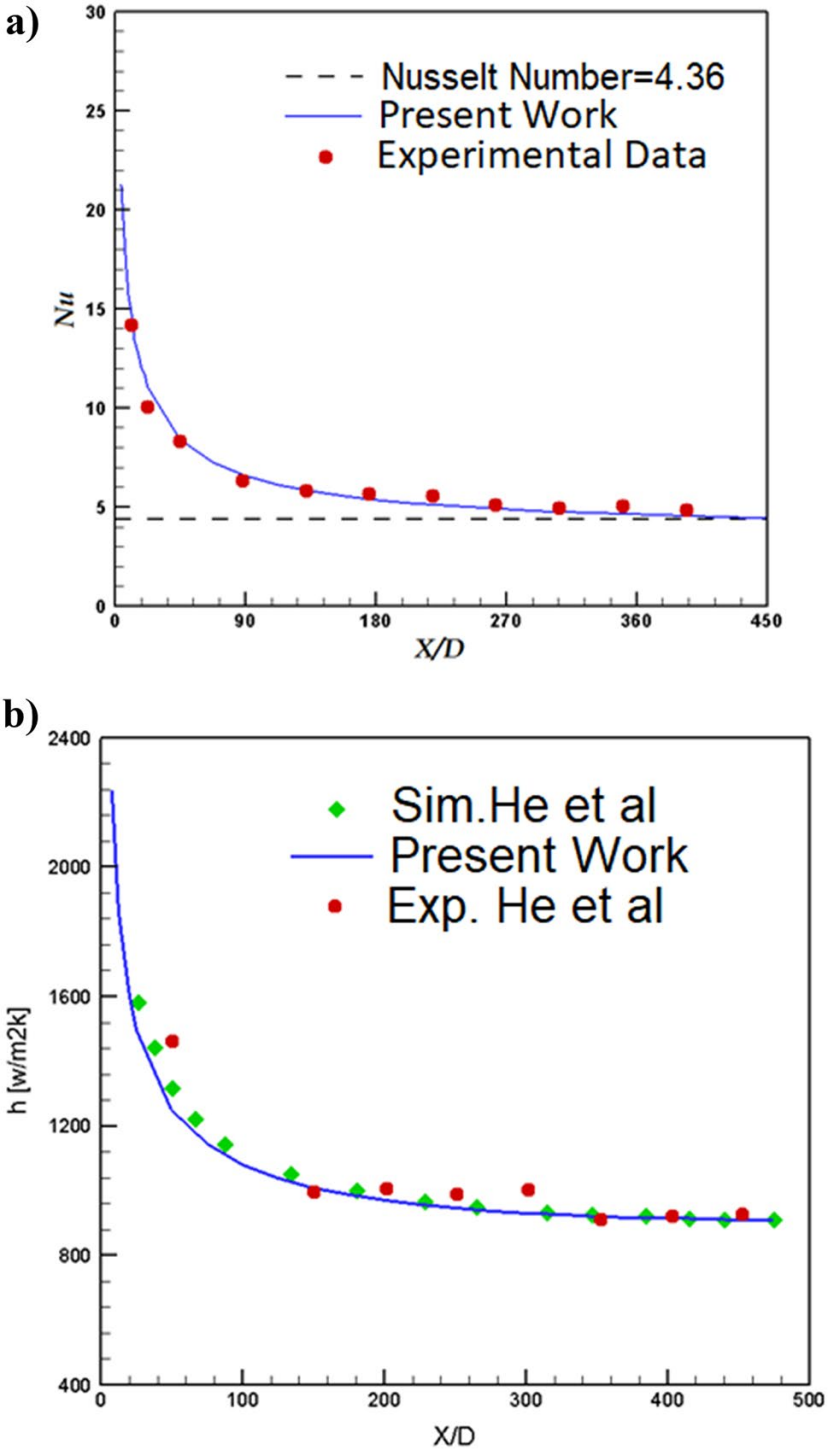
Validation^1^.

Comparison of the achieved results with experimental work is known as validation and it is significant step in the computational study and simulations. The heat transfer analysis of the pure water (Re = 1620) in single tube with constant heat flux are done and presented in Fig. 5a. Our comparison indicates that our results agree well with that of Kim et al.^41^. Comparison of the selected model in presence of TiO2 nanoparticles (24%) are also done with experimental data of He et al.^42^ (Fig. 5b). It is found that deviation of our results with experimental study is less than 7% and it is good agreement.

**Figure 6:**
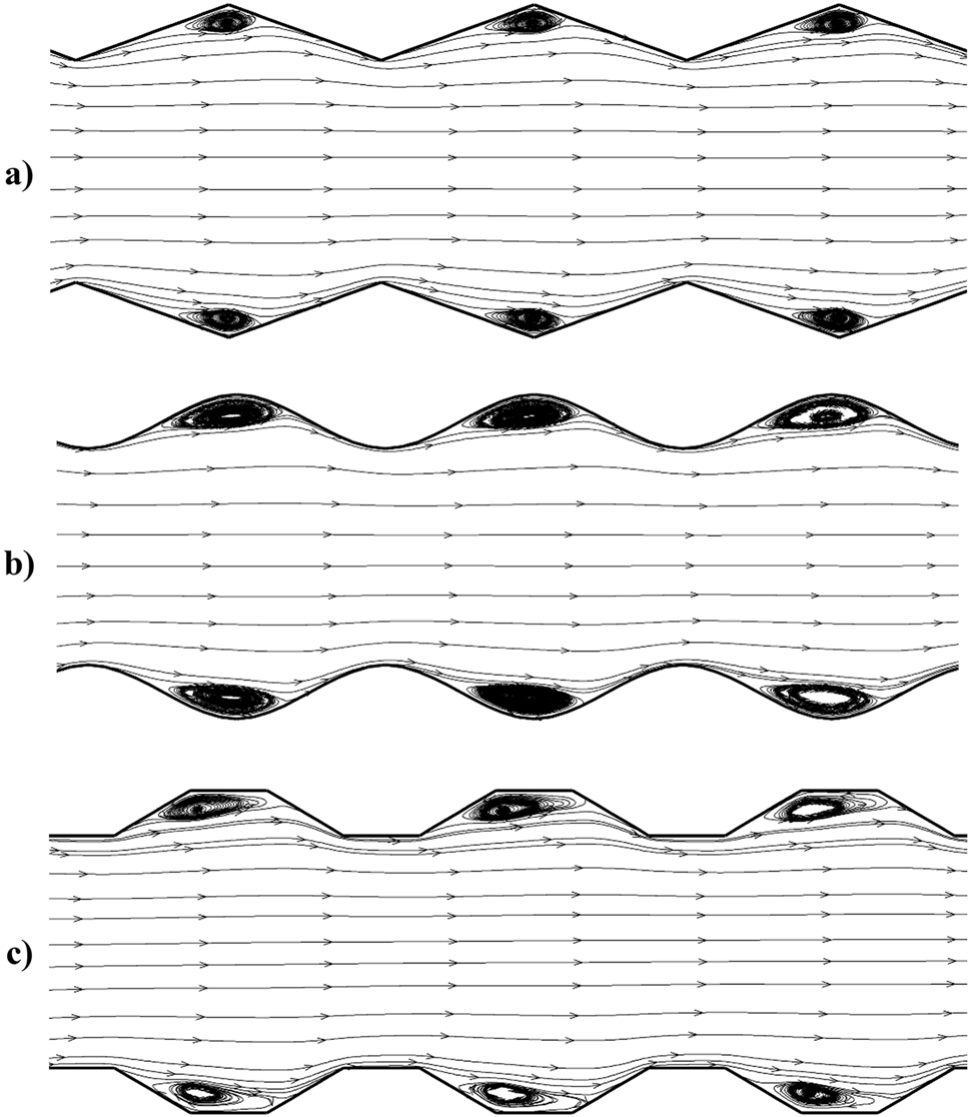
Comparison of streamline.

Comparison of the nanofluid streamline without magnetic field for the selected models are presented in Fig. 6. The circulations are produced in existence of the cavity inside the domain. The size of the circulation is pronounced in the model with sinusoidal wall. The formation of these circulations results in the separations which augment the heat transfer between wall and nanofluid stream.

**Figure 7:**
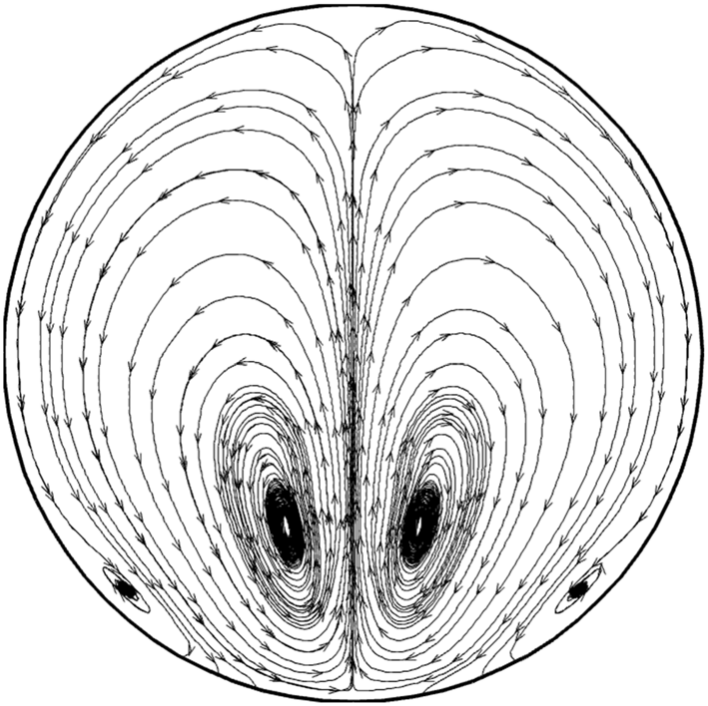
The flow stream in the mid-section of the tube in existence of the magnetic field.

**Figure 8:**
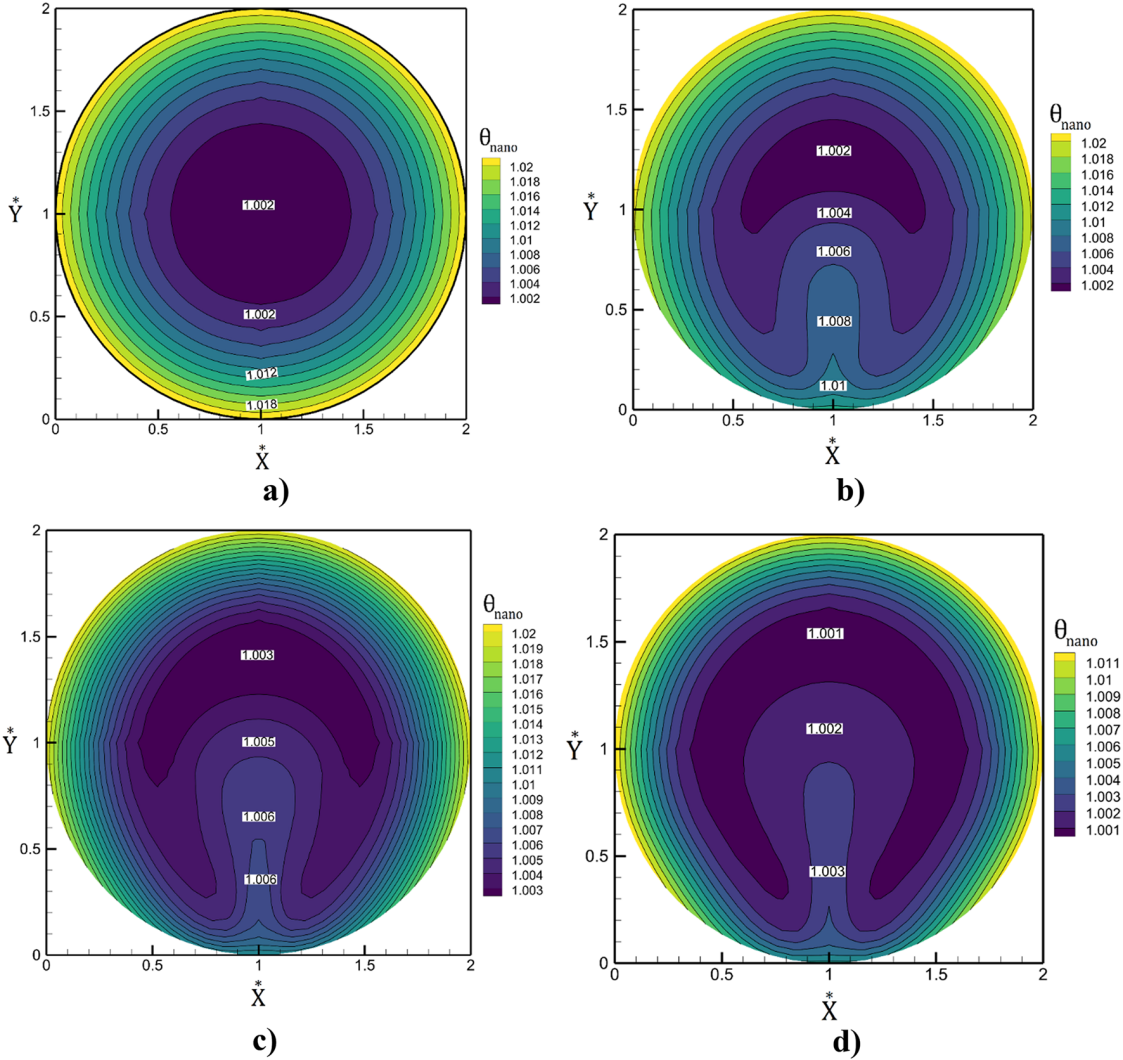
Contour of non-dimensional temperature in the mid-section of tube (**a**) Mn = 0, (**b**) $Mn=1.088\times {10}^{6}$, (**c**) $Mn=2.22\times {10}^{6}$, (**d**) $Mn=3.47\times {10}^{6}$.

The influence of the non-uniform magnetic field on the structure of the nanofluid streamline is demonstrated in Fig. 7. As noticed in the figure, the circulation is split into two sub-circulations which would increase the heat transfer. The influence of the magnetic field intensity on the variation of the temperature inside the smooth tube are displayed in Fig. 8. In this model, Reynolds number of the nanofluid stream and air are 80 and 1500, respectively. It is found that the increasing of magnetic intensity enhances the thermal boundary layer into the center of the tube. Besides, the impact of the streamline is more pronounced on the temperature variation on the cross-section of the tube.

**Figure 9:**
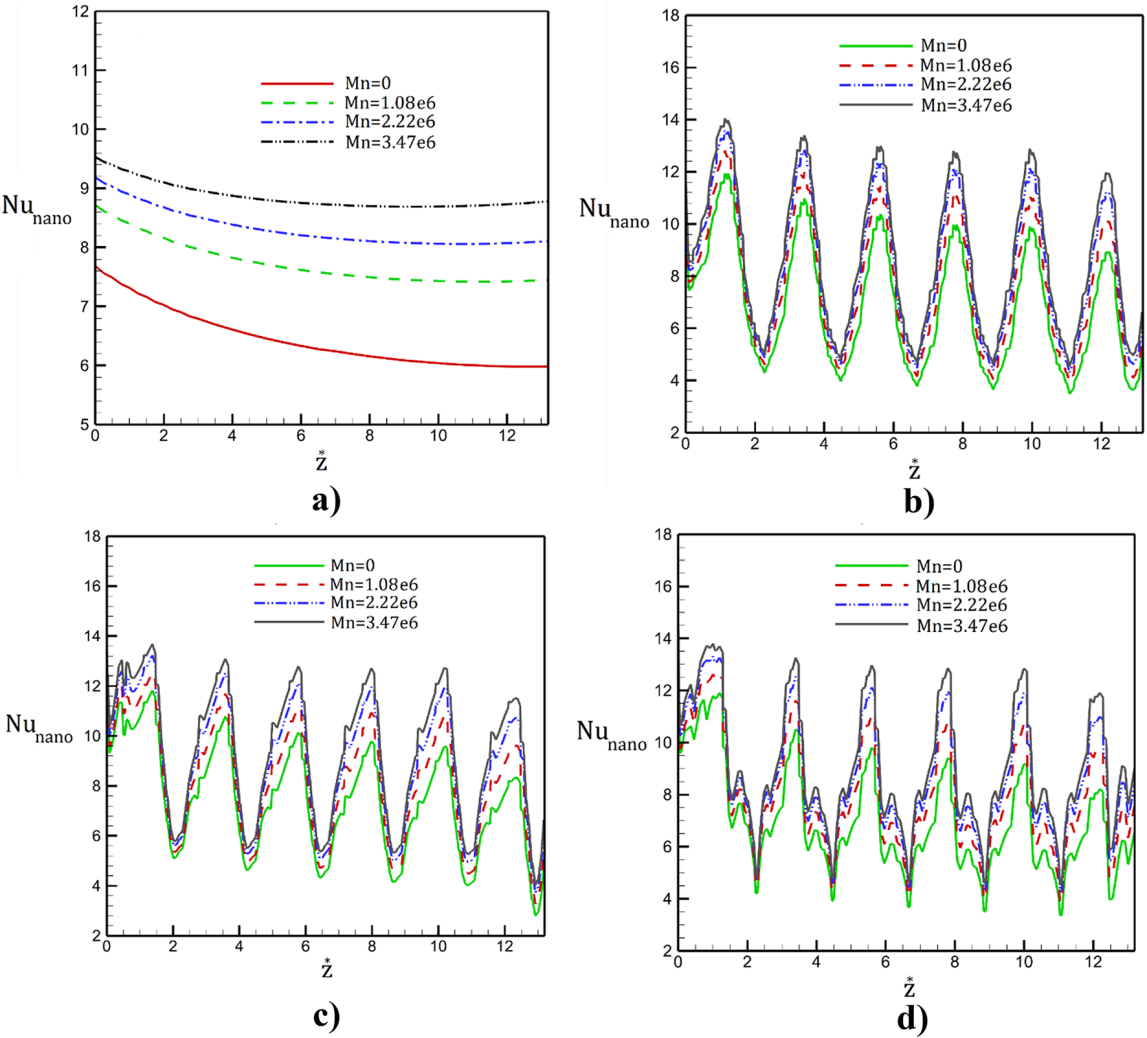
Distribution of the Nusselt number along (**a**) simple, (**b**) sinusoidal, (**c**) triangular, (**d**) square tube in presence of different magnetic intensities.

Comparison of the Nusselt number along the selected geometries under effects of non-uniform magnetic force with different intensities are displayed in Fig. 9. It is observed that the variation of the heat transfer is directly proportional with the shape and size of the circulation in these cavities. Besides, the intensity of the magnetic field enhances heat transfer inside the tube. Comparison of the maximum and minimum value of Nusselt number shows that the sinusoidal and square shapes have highest fluctuation in the heat transfer. It is also observed that application of magnetic field with Mn = 3.47e6 increases the maximum local heat transfer up to 30%.

**Figure 10:**
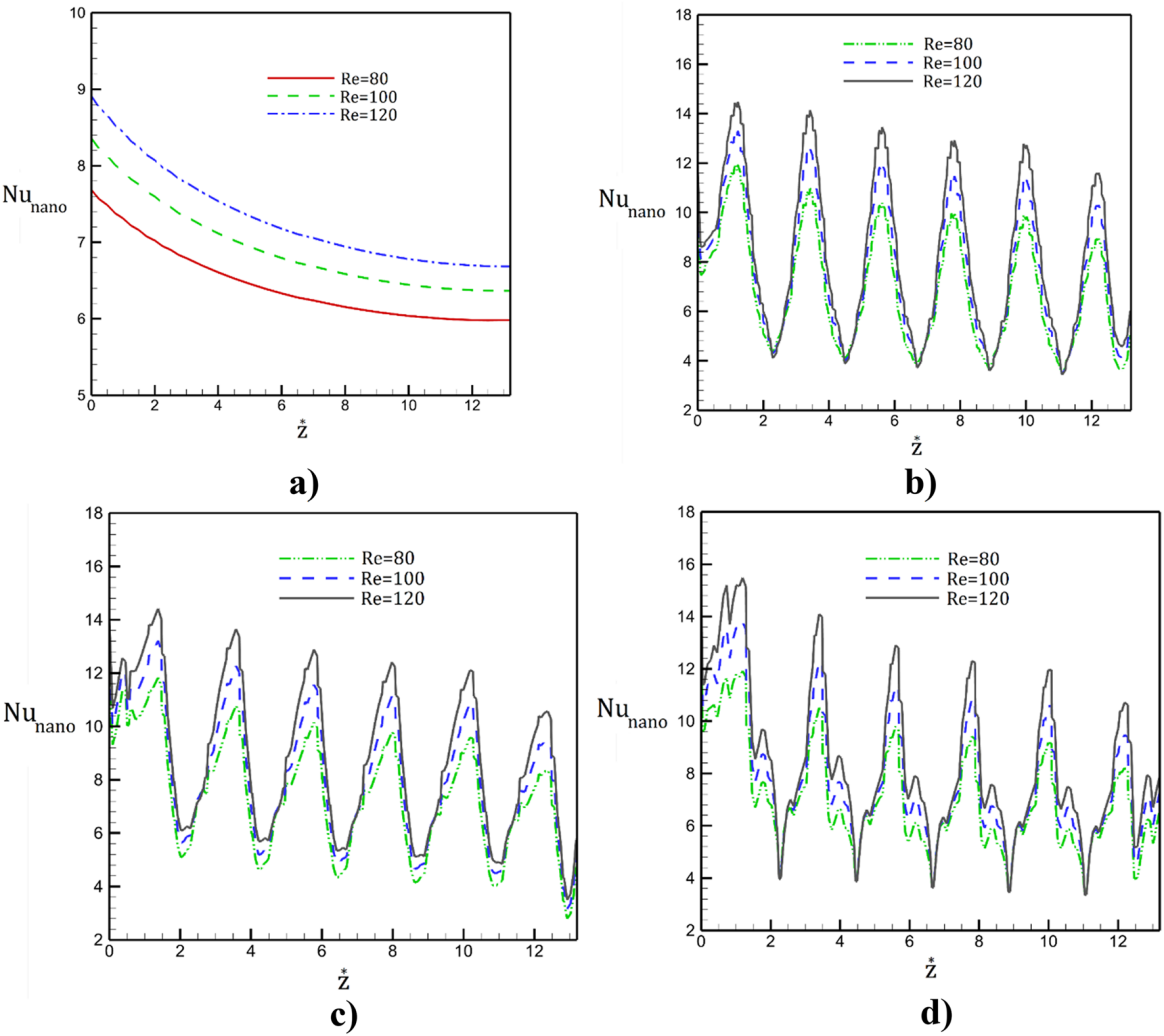
Distribution of the Nusselt number along (**a**) simple, (**b**) sinusoidal, (**c**) triangular, (**d**) square tube in presence of different inlet velocities.

The influence of the inlet Reynolds number on the local Nusselt number are plotted in Fig. 10. Obtained results shows that the heat transfer rate is reduced with periodic cycles along the tube. The maximum heat transfer value happens in the section with lower area while minimum value noticed in higher section area. In fact, this is mainly because of the higher velocity of nanofluid in the section with lower surface area. The impacts of inlet velocity are noticeable in maximum Nusselt number. Meanwhile, in the high Reynolds number, the location of the initial separation moves into the upstream.

**Figure 11:**
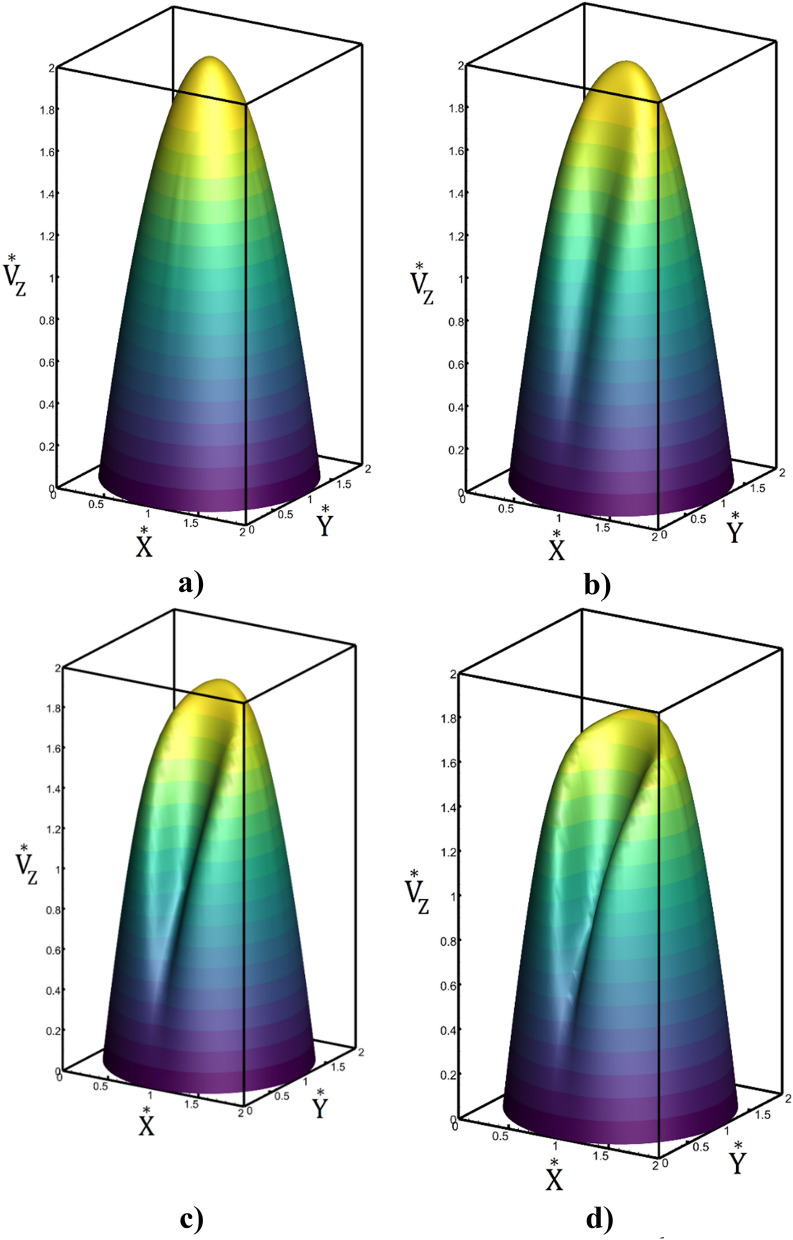
Contour of velocity in the mid-section of tube (**a**) Mn = 0, (**b**) $Mn=1.088\times {10}^{6}$, (**c**) $Mn=2.22\times {10}^{6}$, (**d**) $Mn=3.47\times {10}^{6}$.

The influence of magnetic field on velocity distribution in the mid-section of tube are demonstrated in Fig. 11 for Re_n_ = 80 and Re_i_ = 1500. Achieved contour indicates that velocity distribution become more uniform and nanofluid velocity near wall increases when intensity of the magnetic field is high. Besides, maximum velocity value in the center of tube is decreased in high magnetic intensity.

**Figure 12:**
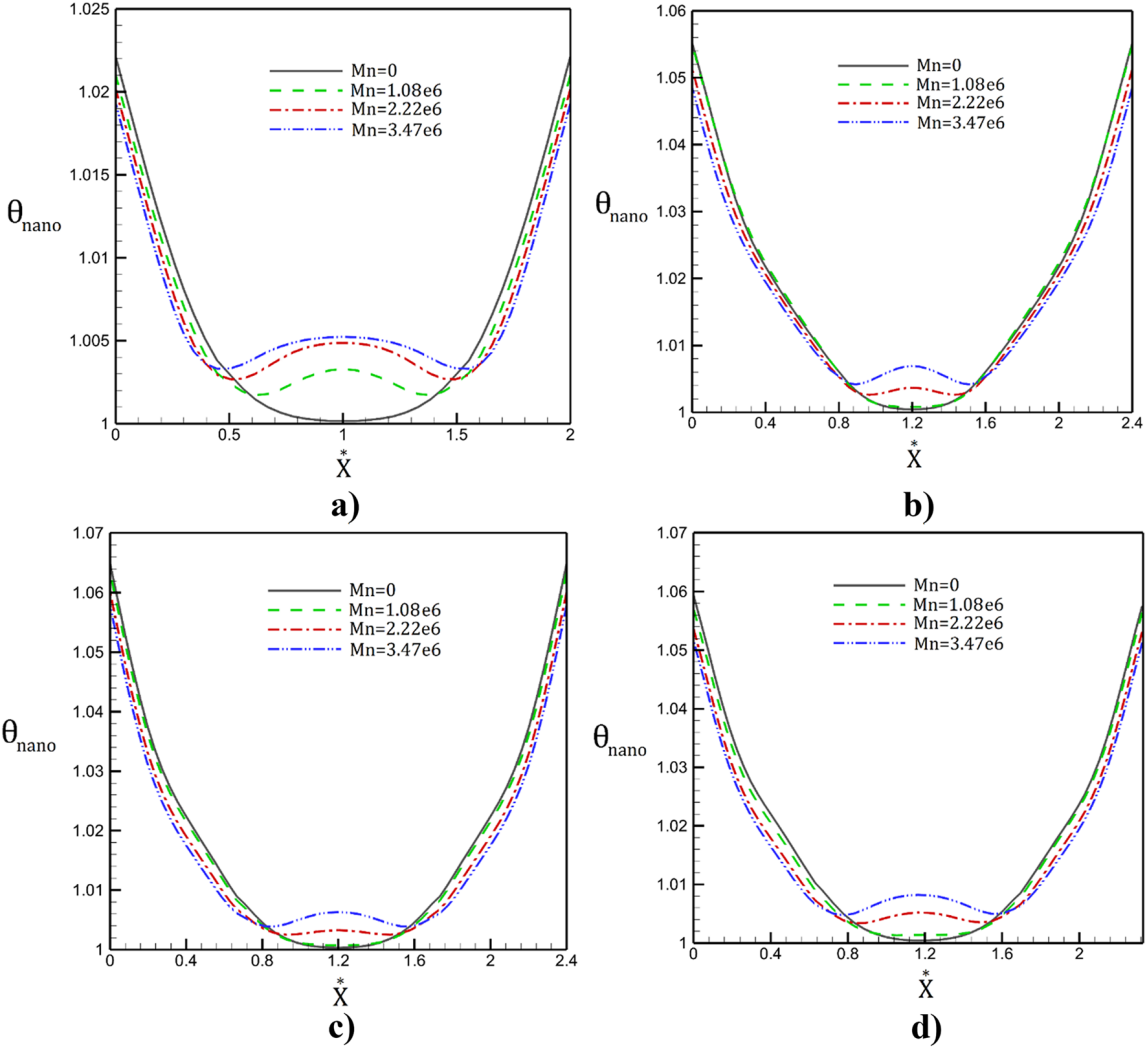
Radial distribution of the temperature (**a**) simple, (**b**) sinusoidal, (**c**) triangular, (**d**) square tube in presence of different magnetic fields.

**Figure 13:**
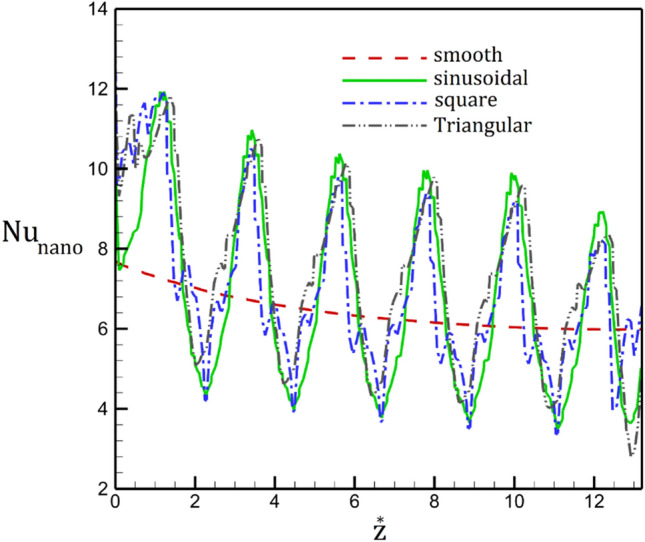
Comparison of Nusselt number along the tube without magnetic field.

The temperature variation of the nanofluid flow for different magnetic intensities and tube shapes are illustrated in Fig. 12. The variation of the temperature indicates that the main impact of the magnetic field is on the temperature near the center of the tube. Besides, temperature value is considerably increased by changing smooth tube with deformed tube (square, sinusoidal and triangular tubes). Variation of the Nusselt number along the tube for different geometries without magnetic field (Fig. 13) also confirm this finding.

**Figure 14:**
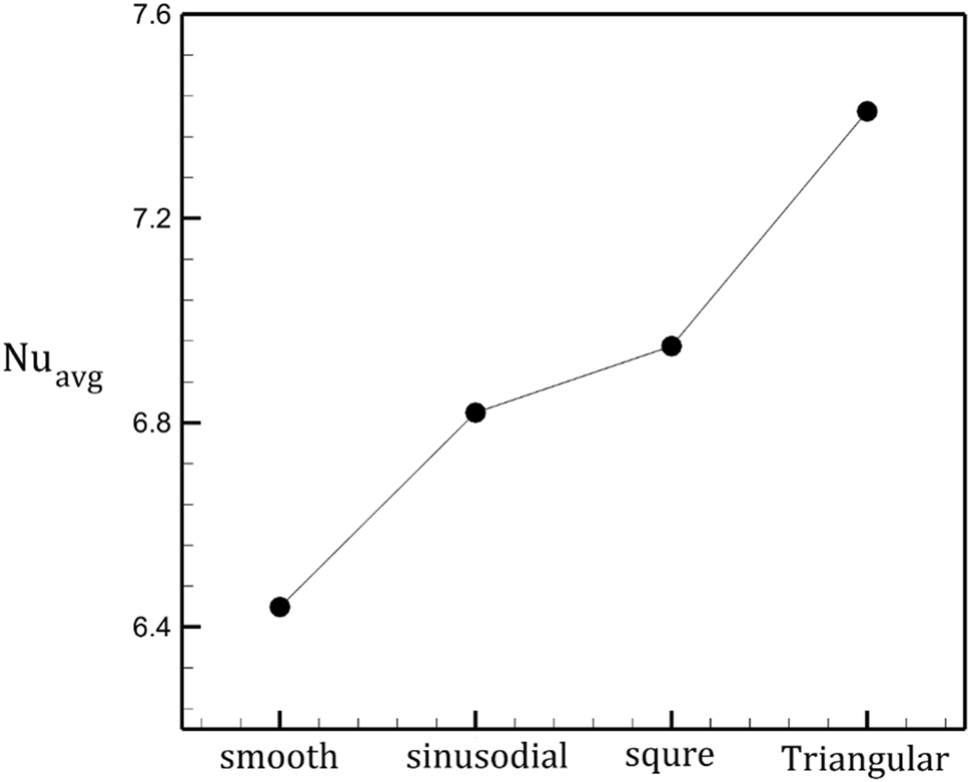
Evaluation of average Nusselt number for different types of tube walls.

Heat transfer performance of the selected geometries are compared in Fig. 14. The variation of average Nusselt number for these configurations indicates that the triangular tube has is more efficient than other configurations. Average Nusselt Number of this model is 15% more than smooth tube.

## Conclusion

In the present work, the influence of the tube profile on the heat transfer performance of the nanofluid flow inside the inner tube with existence of non- uniform magnetic field is fully investigated. This research tried to present the main mechanism of heat transfer by analysis of flow structure and boundary layer distribution inside the tube. For simulation of the nanofluid flow, computational technique is used by solving RANS equations with additional source term associated with the magnetic field of wire. Due to non-uniformity of the magnetic field, the addition of source term is done in both x and y direction. Effects of magnetic intensity and inflow velocity on the average and local Nusselt number are fully investigated. Three shapes of tube wall (sinusoidal, square and triangle) are investigated in this work. Our investigation shows that the production of the circulation inside the cavity of tube plays key role on the local heat transfer, Comparison of the shape of tube indicates that the thermal efficiency of the tube with triangular shape is more than other configurations and its performance is 15% more than smooth tube.

## Data Availability

All data generated or analysed during this study are included in this published article.
